# The Effect of Tourniquet Usage and Anesthesia Method on Prognosis in the Treatment of Dorsal Wrist Ganglion Cysts by Open Surgery

**DOI:** 10.7759/cureus.5981

**Published:** 2019-10-24

**Authors:** Abuzer Uludağ, Hacı Bayram Tosun, Mehmet Mete Yasar, Abdussamed Gunay, Bilge Aydin Turk, Öznur Uludağ

**Affiliations:** 1 Orthopaedics, Adiyaman University Faculty of Medicine, Adiyaman, TUR; 2 Orthopaedics, Istanbul Medipol University, Istanbul, TUR; 3 Pathology, Adiyaman University Faculty of Medicine, Adiyaman, TUR; 4 Anesthesiology and Reanimation, Adıyaman University Faculty of Medicine, Adıyaman, TUR

**Keywords:** ganglion, recurrence, tourniquet, anesthesia

## Abstract

Introduction

Ganglion cysts are the most common soft tissue masses seen on the wrist, which often cause pain or cosmetic complaints. The treatment of these masses includes intra-cystic injections or surgery. Recurrence rates are very high in surgical or non-surgical treatment. Inadequate excision for recurrence after surgery is blamed; however, the reasons for the recurrence still remain mysterious.

Objectives

In this study, the effect of anesthesia selection and tourniquet use on the dorsal wrist ganglion cysts in open surgery was investigated.

Materials and methods

Patients with dorsal wrist ganglion cysts, who were operated with open surgery between 2015 and 2018 and who had at least six months after the surgery, were examined. The patients were divided into two groups: patients who underwent surgery without tourniquet with local anesthesia and patients operated with tourniquet with general or regional anesthesia. Age, sex, cause of operation, visual analog scale (VAS) scores before and after surgery, limitation of movement, postoperative complications, and recurrence were compared.

Results

There was no significant difference between the groups in terms of causes of surgery, recurrence rates, preoperative and postoperative limitations of movement, and complications. In terms of age, the group operated with local anesthesia and without tourniquet was significantly larger. There was also no significant difference between the groups in terms of preoperative pain. Postoperative pain was significantly less in the group operated by tourniquet with general-regional anesthesia.

Conclusion

There is no significant difference in the recurrence and complications between patients operated under local anesthesia without tourniquets and patients operated with tourniquets under general or regional anesthesia during the open excision of the dorsal wrist ganglion cysts. However, it should be kept in mind that postoperative pain does not diminish in later ages, especially in cases of ganglion cysts, and other pathologies may also potentially cause pain in the wrist.

## Introduction

Approximately 60% to 70% of the ganglion cysts are found in the dorsal part of the wrist. Dorsal wrist ganglion cysts are the most common benign structures of the wrist. Wrist ganglion cysts, 1-2 cm in size, often causing pain and cosmetic problems can pass 49% without any treatment. There are three main approaches to treatment: observation, aspiration, and surgery. All treatment approaches have high recurrence rates. Patients treated with aspiration had a mean recurrence rate of 59%, whereas this rate decreased in patients undergoing open surgery. Among the causes of recurrence, inadequate resection has been blamed; however, as the causes of recurrence cannot be fully elucidated, current treatment options remain inadequate [1-4].

The aim of this study was to investigate the effect of anesthesia and tourniquet choice on prognostic factors in patients who underwent open surgical excision of the dorsal wrist ganglion cysts.

## Materials and methods

This retrospective study was conducted at the Adiyaman Training and Research Hospital, Adiyaman, Turkey. The study approval was obtained from the local ethics committee of the Adiyaman University (Approval no: 2018 / 9-12). The patients who underwent open surgery of dorsal wrist ganglion cysts between 2015 and 2018 and who had at least six months after the surgery were evaluated.

Patients whose information could not be reached, patients younger than 14 years of age, and patients who were not compatible with ganglion cysts due to pathology were excluded from the study. Patients were divided into two groups: those operated without tourniquets with local anesthesia and those operated with tourniquets and general or regional anesthesia. Patient files and pathology results were analyzed retrospectively. Age, sex, cause of surgery, visual analog scales (VAS) scores before and after surgery, limitation of movement, complications after and after surgery and recurrence were recorded.

Surgical procedure

**Figure 1:**
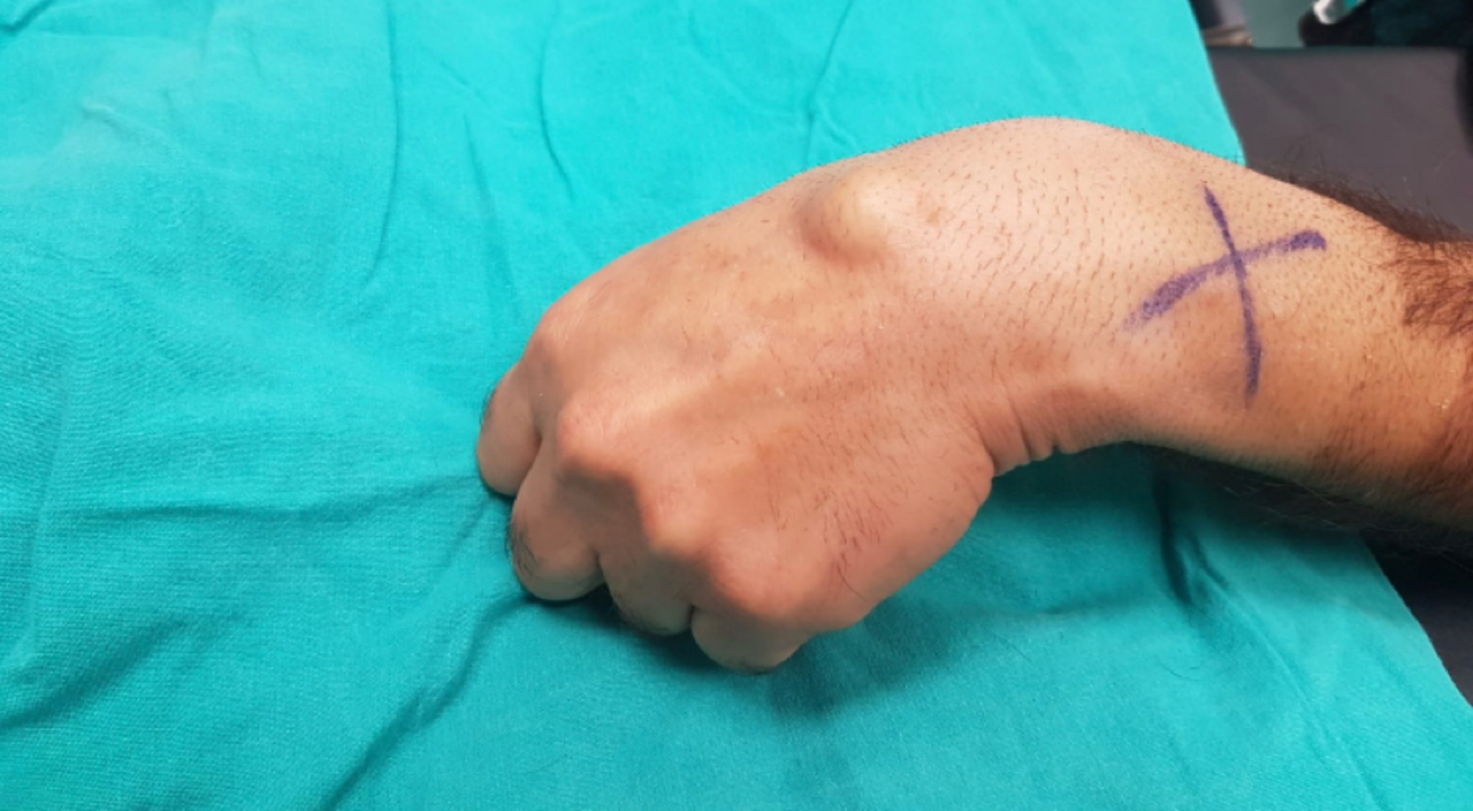
Clinical image of dorsal wrist ganglion cysts

**Figure 2:**
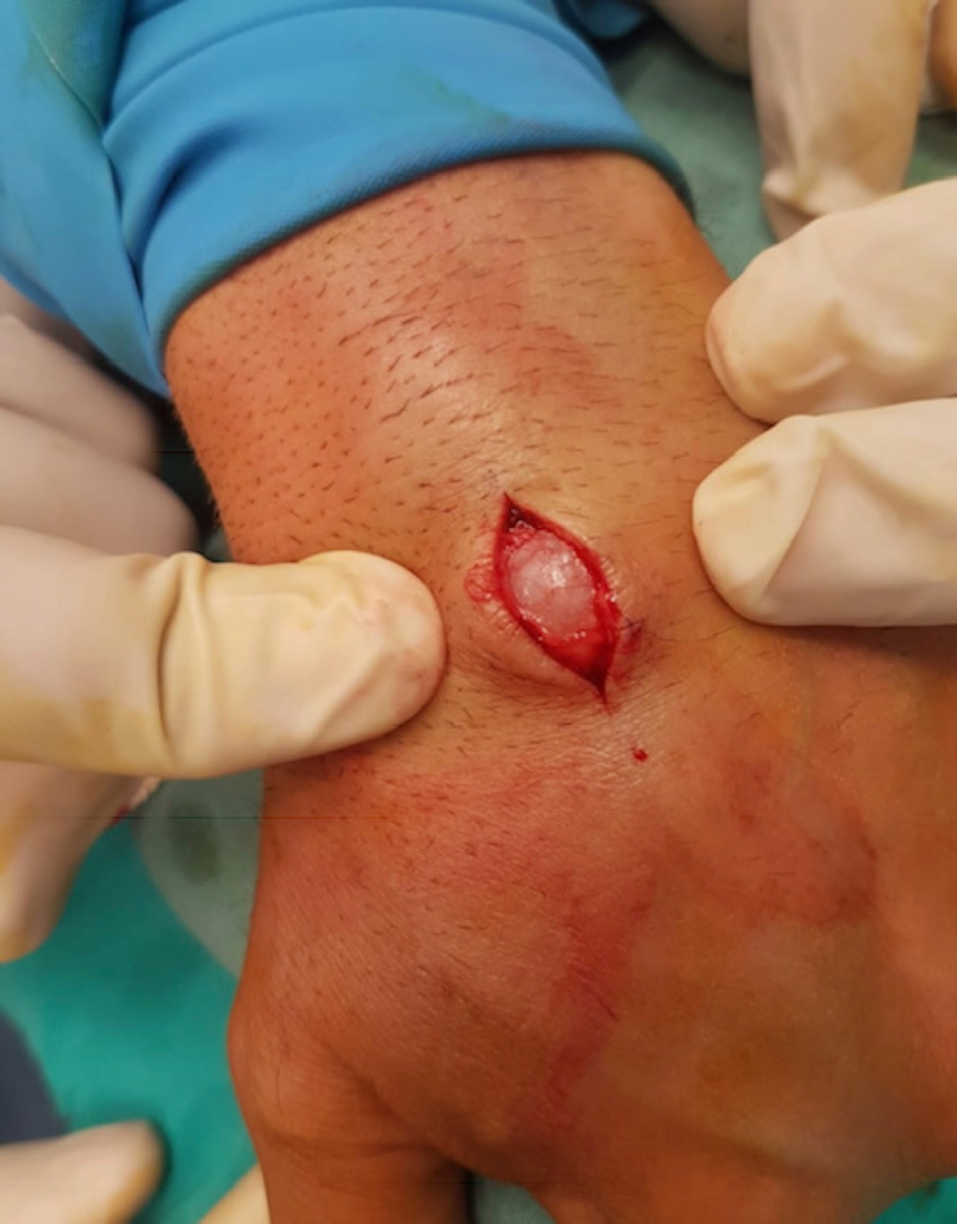
Image of surgical approach with a longitudinal incision

Regardless of anesthesia and tourniquet application, a longitudinal incision was made over the ganglions in all patients. Ganglions were explored to reach the capsule extension. Ganglion cysts were removed with some capsules and capsule repair was performed (Figures 1-2).

Statistical analysis

The data were evaluated with SPSS 21.0 software (IBM, Armonk, NY, USA). In the analysis of variables, the Mann-Whitney U test and Kruskal-Wallis test were used for non-parametric tests. *p* < 0.05 was accepted as significant.

## Results

Between 2015 and 2018, 59 out of 74 patients who were operated on the dorsal wrist with a preliminary diagnosis of ganglion cysts were included in the study. Among the 59 patients, 39 (66.10%) were females and 20 (33.90%) males. The age (mean ± SD) value of the included patients was 30.92 ± 13.39 years. The mean follow-up period was 37.5 months (minimum: eight, maximum: 60; Table 1).

**Table 1:** General distribution of patients operated for dorsal wrist ganglion cysts

Categories		No. (%)
Number of Patients	59	
Age	30.92 ± 13,39 years	
Gender	Female	39 (66%)
	Male	20 (34%)
Affected Side	Right	26 (44%)
	Left	33 (56%)
Cause of Surgery	Pain	29 (49%)
	Cosmetic	16 (27%)
	Pain and Cosmetic	14 (24%)
Complications	Limitation of movement	8 (14%)
	Scatris	5 (8%)
	Hypoesthesia	10 (17%)
	Recurrence	27(46%)

The mean age of the 21 patients (15 women and six men) operated under local anesthesia without tourniquet was 36.05 ± 17.93 years. The mean follow-up time was 37.9 months (minimum: nine, maximum: 60). The lesion sites were dominant hand in eight (38.1%) patients and non-dominant hand in 13 (61.9%) patients. Sixteen (76.1%) patients had preoperative pain. Eleven (52.4%) patients were operated primarily due to pain, five (23.8%) patients were operated for cosmetic reasons, and five (23.8%) patients were operated for pain and cosmetic reasons. The mean preoperative VAS score was 5.7 and 3.0 postoperatively. Preoperative wrist movements were limited in 11 (52%) patients. Two patients had scatris at the wound site. Four patients had hypoesthetic complaints on the dorsal hand. Recurrence occurred in nine patients (42.8%; Table 2).

**Table 2:** General distribution of patients operated under local anesthesia without tourniquet VAS, visual analog scale

Categories		No. (%)
Number of Patients	21	
Age	36.05 ±17.93 years	
Gender	Female	15 (71%)
	Male	6 (29%)
Effected side	Right	8 (38%)
	Left	13 (62%)
Preoperative VAS score	5.7	
Postoperative VAS score	3.0	
Cause of Surgery	Pain	11 (52%)
	Cosmetic	5 (24%)
	Pain and Cosmetic	5 (24%)
Complications	Limitation of Movement	5 (24%)
	Skatris	2 (10%)
	Hypoesthesia	4 (43%)
	Recurrence	9 (19%)

The average age of the 38 patients, who were operated by tourniquet with general-regional anesthesia and consisted of 24 (63.2%) females and 14 (36.8%) males, was 28.08 ± 9.16 years. The mean follow-up period was 37.3 (minimum: nine, maximum: 60) months. The lesion was seen in 18 (47.4%) patients in the dominant hand and 20 (52.6%) patients in the non-dominant hand. Twenty-seven (71%) patients had preoperative pain. Eighteen (47.4%) patients were operated primarily due to pain, 11 (28.9%) patients were operated for cosmetic reasons, and nine (23.6%) patients were operated for pain and cosmetic reasons. The mean preoperative VAS score was 5.73 and 1.55 after surgery. There were 25 (65%) patients in preoperative wrist limitation and three (7%) patients with limited mobility in the postoperative period. In three (7.9%) patients, scatris was seen at the wound site, while six (15.9%) patients had hypoesthetic complaints. Recurrence occurred in 18 (47.3%) patients (Table 3).

**Table 3:** . General distribution of patients operated by tourniquet under general/regional anesthesia VAS, visual analog scale

Categories		No. (%)
Number of Patients	38	
Age	36.05 ±17.93 years	
Gender	Female	24 (63%)
	Male	14 (37%)
Effected Side	Right	18 (47%)
	Left	20 (53%)
Preoperative VAS score	5.73	
Postoperative VAS score	1.55	
Cause of Surgery	Pain	18 (47%)
	Cosmetic	11 (29%)
	Pain and Cosmetic	9 (24%)
Complications	Limitation of movement	3 (8%)
	Scatris	3 (8%)
	Hypoesthesia	6 (16%)
	Recurrence	18 (47%)

There was no significant difference between the groups in terms of the causes of surgery, recurrence rates, presence of preoperative and postoperative limitations of movement, and complications (*p* > 0.05). In terms of age, the group operated without local anesthesia and tourniquet was significantly larger (*p* < 0.05). In addition, there was no significant difference between the groups in terms of preoperative pain, but postoperative pain was significantly less in the general/regional anesthesia group (*p* < 0.05).

## Discussion

Treatment of ganglions is based on the removal of the cyst with the duct of an approximately 1 cm joint. Recurrence of the ganglion cysts is the most commonly reported failure. Although recurrence is accepted as the inadequate excision of the cyst, the reason for recurrence despite good surgical excision could be attributed to other associated factors [4-7]. Recurrence rates vary between 1% and 50% after surgical removal of the ganglion cysts. The differences in the rates of recurrence among studies are the result of heterogeneity such as differences in the number of patients in the studies, changes in the follow-up periods, and the use of different surgical techniques. In our study, 59 patients had a high rate of 46% recurrence. Kulinski et al. failed to repair the capsule after 1 cm of capsule excision and left it open [5]. We also performed capsule repair by surgically removing the base of the cyst with the capsule and considered capsular resection within the capsule repair. Therefore, we presume that the high recurrence rates could be attributed to the increase in the probability of the residual mass after surgery.

Kulinski et al. in their study involving 198 patients with dorsal wrist ganglion cysts reported that gender and age had no effect on the recurrence rates, and relapse was independent of the demographic characteristics of the patients [5].

Some complications such as infection, poor-looking scar, keloid tissue, postoperative limitation of motion, and difficulty in grasping may be observed after the surgical removal of the dorsal wrist ganglion cysts. These complications have reached 20% in some studies [8].

Movement limitation of the wrist is an important complication observed after the excision. Aydın et al. showed stiffness of the wrist in five (12.5%) out of 40 patients as a result of the excision of the dorsal wrist ganglion cysts [9]. Dermon et al. reported a 4.5% stiffness in the wrist [10]. In our patients, movement restriction was observed in eight patients (13.5%) postoperatively. Long-term immobilizations, delayed rehabilitation programs, and improper closure of the joint capsule during excision of the cyst could contribute to the movement limitation [9-12].

Pain, cosmetic reasons and weakness are among the reasons for patients' acceptance of the surgical removal of the dorsal wrist ganglions. Pain is an indication for surgical treatment of the dorsal wrist ganglion cysts and is thought to result from the compression of the terminal branches of the posterior interosseus nerve [13]. Studies suggest 46% to 79% of the patients underwent surgery due to the pain caused by the dorsal wrist ganglion cysts [10,12,14]. Similarly, in this study, 49% of the patients underwent surgery because of isolated pain, 27% due to cosmetic reasons, and 24% due to isolated pain and cosmetic purposes. Although ganglion cysts may be a direct cause of pain, it should also be considered pain could be of intra-articular and extra-articular origin. Osterman et al. reported that 42% of the ganglion cysts could be accompanied by scapholunate (SL) ligament damage, triangular fibrocartilage complex (TFCC) lesions, and radial and triquetral cartilage injuries [15]. Kim et al. reported that patients with wrist carpal instability had increased pain after cyst excision [16]. Evidently, the lesions associated with this ganglion are more likely to develop with age. In our study, it is noteworthy that postoperative pain did not decrease with advanced age. This suggests a secondary pathology that causes pain in the wrist in the elderly.

Iatrogenic sensory or motor nerve injuries vary depending on the localization of the ganglion and are often transient. Careful surgical planning and expiration may reduce this rate of replication. Hwang et al. in their study involving 22 patients observed 4.5% transient neuropraxia [17]. In our study, 17% had neuropraxia.

To the best of our knowledge, there is no study on the use of tourniquets or the choice of anesthesia for the treatment of dorsal wrist ganglion cysts. In our study, the choice of anesthesia and tourniquet use did not make a significant difference in the recurrence and complications. However, if we do not have contraindications, we believe that patients should undergo surgery using tourniquets under general or regional anesthesia.

The retrospective nature of this study and the small sample size can be considered its major limitations.

## Conclusions

The use of tourniquets or choice of anesthesia has no effect on the recurrence of dorsal wrist ganglion cysts and has similar characteristics in terms of complications. However, it should be kept in mind that carpal instability or other pathologies such as joint or extra-articular, especially with advancing age, could potentially cause pain in the wrist.
